# Seasonal dynamics of fine root length in European beech: unveiling unexpected winter peaks and summer declines

**DOI:** 10.1007/s00442-025-05670-y

**Published:** 2025-02-07

**Authors:** Aron Garthen, Kirsten Brandt, Marcin Klisz, Andrey V. Malyshev, Bo Peters, Robert Weigel, Juergen Kreyling

**Affiliations:** 1 Experimental Plant Ecology, Institute of Botany and Landscape Ecology, University of Greifswald, Soldmannstraße 15, 17489 Greifswald, Germany; 2 Department of Silviculture and Genetics of Forest Trees, Forest Research Institute, 05-090 Raszyn, Poland; 3 Ecological-Botanical Garden, University of Bayreuth, 95447 Bayreuth, Germany; 4 Plant Ecology, University of Goettingen, 37073 Goettingen, Germany

**Keywords:** Belowground, *Fagus sylvatica* L., Minirhizotron method, Temperate forest, AI root detection

## Abstract

Fine roots play a crucial role in many ecological and biogeochemical processes in temperate forests. Generally, fine root biomass is expected to increase during the growing season, when water and nutrient demands are high, but information on seasonal variability is still scarce. Here, seasonal differences in root length of European beech (*Fagus sylvatica* L.) were analysed at eight sites within its north-eastern distribution range. Fine roots of mature trees were monitored using minirhizotrons. Scans were taken for three different depths at the beginning of winter, the end of winter and over the summer for two consecutive years, and analysed automatically by an AI-algorithm (RootDetector). An additional experiment was carried out to show that the RootDetector was unaffected by changes in soil moisture. Root-length density was 40% higher at the beginning of winter and 51% higher at the end of winter than in summer. Our results indicate a net root loss during adverse conditions in early summer, but no trend towards deeper root growth over these drier periods. Interestingly, the root loss was compensated afterwards during more favourable conditions in autumn. We could show that fine root length in temperate forests is seasonally more variable and, so far, less predictable than previously assumed. A profound understanding of this seasonal variability is important for modelling terrestrial biogeochemical processes and global carbon fluxes.

**Supplementary Information:**

The online version contains supplementary material available at 10.1007/s00442-025-05670-y.

## Introduction

Belowground biomass in forest ecosystems plays a fundamental role in the global carbon cycle (Jackson et al. 1997). Roots account for more than 20% of total forest biomass (Ma et al. 2021), and their contribution to net primary production is even higher (Jackson et al. 1997), because of high turnover rates of fine roots (< 2 mm in diameter). In terrestrial ecosystems, fine roots are responsible for acquiring essential soil resources (McCormack et al. 2015). In spite of their high relevance for ecological and biogeochemical processes, information on the temporal dynamics of fine roots is still scarce compared to the aboveground plant organs.

In contrast to shoots, roots do not experience winter dormancy (Fernandez and Caldwell 1975; Malyshev et al. 2023). Belowground growth of temperate broadleaved trees is possible throughout the entire year in temperate broadleaved trees (Resa 1877), but growth of European beech (*Fagus sylvatica* L.) usually ceases if soil temperatures fall below 2.5 °C (Schenker et al. 2014). Above- and belowground phenology are thus not necessarily in-sync with each other (Blume-Werry et al. 2016; Radville et al. 2016; Malyshev et al. 2023). If root production happens during winter or very early in the growing season (e.g. Gaul et al. 2008), growth can be fuelled by carbohydrates that are stored in woody tissues in autumn (Najar et al. 2014). Fine root production is usually reduced during winter (Alvares‐Uria and Körner 2007; McCormack et al. 2014; Schenker et al. 2014) and increases during April in European beech, shortly before the growing season starts (Mainiero et al. 2010). Peaks appear at the time of bud burst in spring and in early summer (Mainiero et al. 2010; Montagnoli et al. 2014). This timing is assumed to balance between carbohydrate availability from photosynthesis and periods of optimal temperatures, water and nutrient supply (Radville et al. 2016). Once established, lifespan of fine roots is highly variable and depends on endogenous factors, but also on external abiotic factors, time of the year and tree constitution (Leuschner 2020). Fine root longevity estimates for European beech range from less than 77 days (Mainiero et al. 2010) to 412 days (Mariën et al. 2021) and even to more than 1000 days under optimal conditions (Meier and Leuschner 2008b). Pregitzer et al. (2000) and Mainiero et al. (2010) found a decrease in average root longevity and an increase in root mortality during the growing season, likely due to higher respiration rates and increased microbial activity at higher soil temperatures. Generally, fine root mortality in temperate forests seems to be more evenly distributed throughout the year than fine root growth.

The development of European beech total fine root biomass and length is a complex result of strong environmental influences on root production and root mortality, with higher root turnover in more stressful environments and little difference between populations (Meier and Leuschner 2008b; Hertel et al. 2013). Beech total root biomass increased along a precipitation gradient in western Germany towards sites with less annual precipitation (Hertel et al. 2013). In a forest within the species’ southern distribution range, total fine root length and standing biomass determined by soil cores peaked in mid-July with a second increase in September (Montagnoli et al. 2014). Length and mass of very fine roots (d < 0.5 mm) was doubled from May to July and followed a second-order polynomial relationship for soil moisture (optimum around 50 vol.%) and soil temperature (optimum 14 °C). Fine root biomass and length also peaked in a *Quercus* forest in July and a second but lower peak was observed in October, whilst values during the winter period were lowest (Montagnoli et al. 2019).

One of the most common methods used for belowground studies is the minirhizotron method (Johnson et al. 2001). In contrast to soil cores and ingrowth cores, it provides a non-destructive option for long-term monitoring of root dynamics (Freschet et al. 2021). As a ground-breaking innovation in recent years, there are now ways to analyse root images of minirhizotrons much faster and more reliably using artificial intelligence with the aim to overcome the logistic limitation of small sample sizes in root research and provide more robust and generalisable results (e.g. Peters et al. 2023). However, the validity of these algorithms is still an ongoing topic and the capabilities and accuracies are continuously being improved. One of the remaining questions is whether the root detection probability is affected if the visual contrast between roots and soil is modified by changes in soil moisture.

To advance such novel techniques of root research and to investigate root dynamics in Central Europe’s most important forest ecosystem type, we analysed seasonal patterns in root length density of European beech at its north-eastern distribution range using minirhizotrons. Root scans were taken from 2021 to 2023 at the beginning of winter, end of winter and in summer at eight sites along a large climatic gradient (∆ mean winter temperature = 4.0 K) between Rostock in Germany and Gdansk in Poland. Root-length density was defined as the root length per scanned area of the minirhizotron tube surface and determined with the RootDetector (Peters et al. 2023). We hypothesised that (1) the root-length density follows a clear seasonal pattern with higher root-length density in the summer compared to the beginning and end of winter. To confirm the validity of our results, we also performed an experiment that tested whether the root detection probability differed with soil moisture, as this was considered to be the most important factor potentially complicating comparison between seasons. We expected that (2) roots are better detectable in wet soils than in dry due to better soil-tube contact and higher visual contrast between the darker soil and mostly bright roots in wetter soil.

## Material and methods

### Field sites and tree selection

**Fig. 1:**
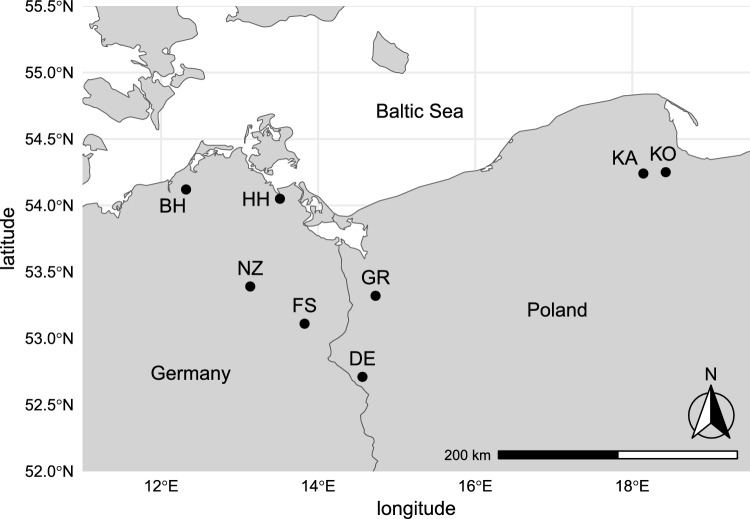
Map of beech forest monitoring sites in Northeast Germany and Northwest Poland

Root length was monitored at eight forest sites dominated by mature European beech located in the Pleistocene lowlands of Northeast Germany and Northwest Poland (Weigel et al. 2021). These sites were distributed along a climatic gradient of 500 km with decreasing winter temperatures from west to east (Fig. 1), covering stands from the central to the north‐eastern distribution margin of European beech (Bolte et al. 2007). The study sites were covered by monospecific beech stands with their typically very sparse and species-poor understory mainly consisting of small-statured spring geophytes and a limited number of small beech seedlings (Weigel et al. 2019), which are almost absent in terms of aboveground biomass at our sampling dates. Therefore, we assume that the roots observed in this study originate predominantly from mature beech trees. Selection of the sites focussed on choosing stands with similar pedological and hydrological conditions. The soil type at all sites was sandy Cambisol with similar soil texture, mostly sandy silt to silty sand (Weigel et al. 2021). The climate at the warmest sites (BH, HH) is characterised by relatively mild winters with long-term means of coldest month mean air temperatures around 0 °C, whilst the eastern sites are below -3 °C (Haylock et al. 2008). Long-term mean annual precipitation ranged from 520 to 650 mm (ESM3). The study winters were warmer than usual, mean coldest month temperatures ranged from  – 1.3 °C to 1.8 °C in winter 2021/22 and from -0.6 °C to 1.0 °C in winter 2022/23 (for further details and average winter temperatures see ESM4). Also, the early growing season between April and June was drier than the long-term means in both study years (precipitation Apr – Jun long-term: 138–158 mm, Apr–Jun 2022: 78–120 mm, Apr–Jun 2023: 59–129 mm), whilst the annual precipitation sums were only slightly lower than usual at most sites (ESM3). A dendrochronological pre-study was carried out to identify three mature beech trees that best represented the growing conditions at each site (Weigel et al. 2018) and one of these was randomly chosen for the root-length monitoring (three-fold replication only at the coldest site KA). Tree diameters ranged from 36 to 49 cm, heights from 29 to 40 m and age from 82 to 181 years, when the study sites were established (Weigel et al. 2018). The gradient design as well as different tree ages across sites allow for general results representing a broad range of beech forest ecosystems at the species’ north-eastern distribution range.

The target trees were fenced to exclude large mammals and six minirhizotron tubes were installed, evenly distributed around each tree at a distance of 2 to 3 m from the trunk in 2016 (NZ site had only three tubes because of rocky underground). Half of the tubes reached 30 cm below the soil surface and half down to 45 cm below the soil surface. About 70% of fine roots occur in the upper 30 cm and fine root density decreases exponentially with soil depth (Meier et al. 2018). We therefore assume that the vast majority of fine roots was captured by our dataset. Minirhizotrons (outside diameter 70 mm) were installed at an angle of 45° tilted away from the sample tree, capped and taped aboveground to prevent light entry. Scans of 216 × 196 mm were taken by CID-600 root scanners (CID Bio-science Inc., Camas, USA) at a resolution of 300 dpi, repeated at two or three non-overlapping depth levels, depending on the total length of the minirhizotrons. Root scans were taken between November 2021 and July 2023 with one measurement at the beginning of the winter (beginning of November) and one measurement at the end of the winter (end of March/beginning of April) as well as one measurement during the subsequent summer (end of June/ beginning of July), respectively, adding up to six measurement dates covering 2 full years. The sampling point in March/ April was chosen to sample the state after winter and before the growing season. Leaf-out of beech usually starts in April (Kolář et al. 2016; Malyshev et al. 2022). July, centred in the growing season was chosen to represent the state right after the peak of tree growth (Strieder and Vospernik 2021; Debel et al. 2024). We expected highest root-length density at this measurement point. November was chosen as sampling point at the end of the growing season, shortly after the leaf shedding of beech in late October (Schieber et al. 2013), and prior to the onset of winter. Due to reduced demand of water and nutrient uptake towards the end of the aboveground growing season and still warm soils, which may promote root decomposition, we expected a net loss of roots at this point in time compared to summer. Taken together, the net change on root-length density between March and July covers the peak of the aboveground growing season, the change between July and November should cover the presumed decline from peak season to start of winter, and the change between November and March covers winter, i.e. the aboveground dormant season.

### Quantification of root-length density with the RootDetector

Root length per scan was determined with the RootDetector, a convolutional neural network (CNN) specifically designed for the automatic detection and segmentation of plant roots in minirhizotron images, as outlined by Peters et al. (2023). Utilising a U-Net architecture, RootDetector demonstrates high reliability in identifying root structures within highly heterogeneous substrates, achieving an F1 score of 0.51 over the 24 validation pictures annotated manually for the given dataset. The F1 score, ranging from 0 to 1, represents the harmonic mean between precision and recall and is a commonly used metric to assess performance of machine learning models. Furthermore, Peters et al. (2023) have shown that RootDetector enables precise total root-length estimations (r^2^ = 0.99 when compared to human experts) by employing a secondary skeletonization algorithm and calculating root length using the formula proposed by Kimura et al. (1999). Here, Kimura root length per scan expressed as mm cm^−2^ was used as root-length density.

### Soil moisture experiment

To determine whether the automated root detection is affected by soil moisture, an experiment was conducted at the Hanshagen (HH) forest site at the beginning of September 2023. Three target trees with six minirhizotrons each were used for the soil moisture experiment and the upper 15 cm of the soil were used for the analyses. First, root scans were taken from all tubes during a dry period at the end of summer and simultaneously the volumetric soil moisture in the upper soil was measured by a Fieldscout TDR 300 Soil moisture meter (Spectrum Technologies, Aurora, USA). Afterwards, the six tubes per tree were randomly assigned to three treatments. The first treatment received 40 L of water on an area of 1 m^2^ around the tube in several doses over 2 h, the second treatment received 80 L and the third group was treated as a reference and was not watered at all. After a waiting period of three hours, which ensured that the soil was evenly moistened but short enough to avoid artefacts caused by freshly growing roots (Guilloy et al. 2011), root scans and soil moisture measurements were repeated.

### Statistical analyses

All statistical analyses were performed using the Software R 4.4.0. (R Core Team 2024) and additional packages listed in the Online Resource (ESM5). To test for differences in root-length density between seasons, ANOVAs were applied for linear mixed-effect models. Mixed model formulation included the season and depth and their interaction as fixed effects and tube nested in site as well as the study year (first study year includes start of winter 2021, end of winter 2022, summer 2022 and the second study year the same for 2022/23) as random effects, thereby accounting for the spatial and temporal dependencies in the data: root-length density ~ season * depth + (1|site/tube) + (1|study year). Parametric assumptions of the linear models (homoscedasticity and normal distribution of residuals) were checked visually by diagnostic plots and improved by square root transformation.

For the moisture experiment, the data were also analysed by ANOVAs of linear mixed effects models. To explore the effect of the water treatment on soil moisture, water treatment (0 L/ 40 L/ 80 L) and session (before vs. after watering) and their interaction were included as fixed effects and tubeID was used as random effect: Soil moisture ~ water treatment * session + (1|tubeID). The same model with root-length density instead of soil moisture as dependent variable was used to search for potential differences in the effect of the water treatments on root-length density. Since actual root-length density is not expected to change much within a few hours, this would indicate differences in root detection probability. For visualisation, the ratio between values after watering and before watering was calculated, so that values deviating from 1 indicate differences between the sessions.

## Results

### Root-length seasonality

**Fig. 2:**
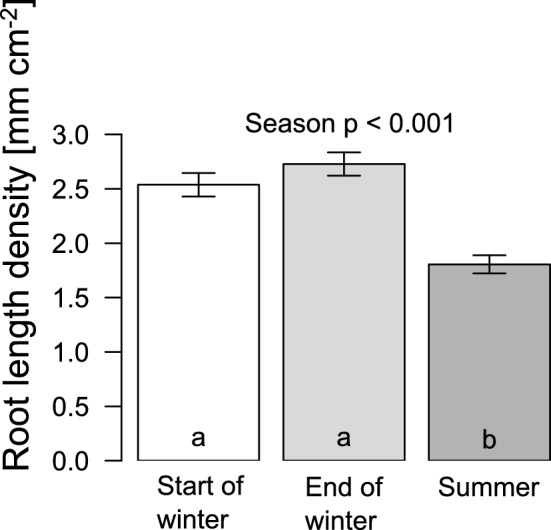
Root-length density in mm per cm^2^ of minirhizotron scans in different seasons from autumn 2021 to summer 2023 across eight forest sites between Rostock and Gdansk, quantified by the AI RootDetector. Shown are mean values and standard errors over 272 minirhizotron scans per bar. The letters displayed in the bars result from the mixed model ANOVA with subsequent pairwise comparison of estimated marginal means

Root-length density was significantly higher at the beginning of winter (2.54 ± 1.77 mm cm^−2^) and the end of winter (2.73 ± 1.78 mm cm^−2^) compared to summer (1.81 ± 1.39 mm cm^−2^; Fig. 2; *p* < 0.001). In relative numbers, root-length density was 40% higher at the beginning and 51% higher at the end of winter than over the summer. The general pattern was consistent amongst study sites (ESM2) and amongst years with a particularly low root-length density for summer 2023 (ESM1). Mean values at the end of winter did not differ from the beginning of winter (*p* = 0.105). The differences between seasons were not affected by rooting depths (interaction season:depth *p* = 0.660), thereby implying no sign of deeper rooting in (dry) summers.

### Moisture experiment

**Fig. 3:**
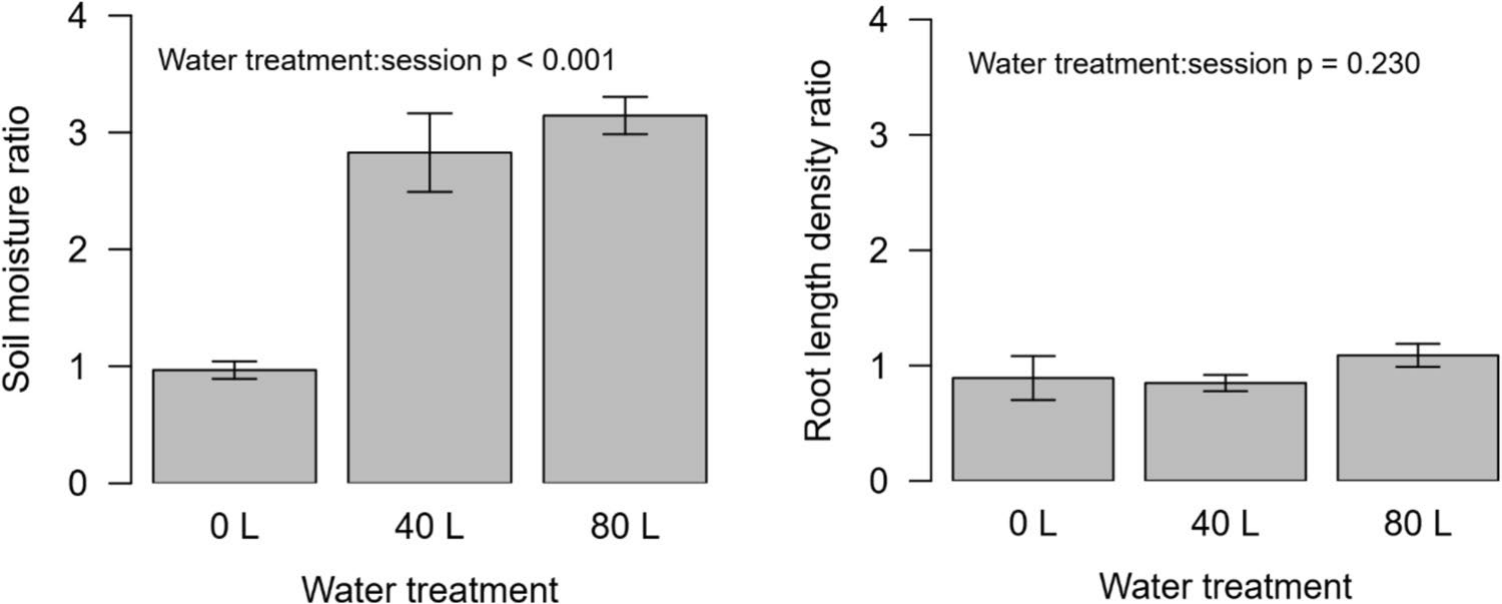
Effect of the water treatment on the volumetric soil moisture (left) and on the root-length density determined by the AI RootDetector (right). Moisture and root-length density ratios were calculated by dividing the values after watering by the values before watering (= ambient conditions). Bars show means and standard errors of six tubes per treatment in the upper 15 cm of the soil

The water treatment significantly increased soil moisture (water treatment:session *p* < 0.001), but did not affect root-length density detection (water treatment:session *p* = 0.230; Fig. 3).

## Discussion

### Seasonal pattern of root length

We found a significantly higher root-length density at the beginning (+ 40%) and end (+ 51%) of winter compared to summer (Fig. 2). This contradicts previous findings, which documented highest fine root length for summer (Montagnoli et al. 2014, 2019). Root decay apparently exceeded production rates during the early growing season, leading to a net loss of roots. One explanation for high decay rates in spring and early summer could be that roots have already died off in winter. Roots of temperate tree species are susceptible to frost events below  – 5 to  – 10 °C (Noshiro and Sakai 1979). Litterbag experiments which we conducted during the same period as the root monitoring showed that decomposition rates were almost 40% lower during winter compared to early summer, so that dead roots may have remained in the soil until spring. However, high winter root mortality is not very likely, because almost no soil frost appeared during the study winters (absolute minimum soil temperature:  – 0.4 °C). Another possible factor might be that precipitation was lower than usual during the early growing season. Precipitation sums between April and June averaged over all sites were 35% lower in 2022 than the long-term means (36% in 2023, ESM3). Temperate trees react to summer drought either by increased root loss to save carbon or by increased growth to maximise water-absorbing surface area (Leuschner et al. 2001; Meier and Leuschner 2008a). For European beech, an increase in fine root biomass has been shown under mild water stress, but under severe drought, growth is usually supressed (Leuschner 2020). At the same time, root mortality increases during extreme drought and increased temperatures, leading to a reduction in living fine root biomass (Pregitzer et al. 2000). Within the analysed upper 45 cm covered by three root images (= 15 cm depth per picture), we did not detect any trend towards more root growth in deeper and presumably wetter soil horizons during the driest period of the year. Whilest several herbaceous plants have been shown to root deeper under drier conditions (Reader et al. 1993), this seems not necessarily be the case for European beech (Meier and Leuschner 2008b; Meier et al. 2018). However, a shift of beech roots from the organic layer towards the upper mineral soil has been documented with decreasing precipitation (Meier and Leuschner 2008a). This effect is potentially also present at our sites within the upper part of the soil (upper picture), but would then only be visible with a finer depth resolution. It is also possible that roots grew below the observed depth of 45 cm. The amount of total biomass or length in deeper soil layers is expected to be low (Meier and Leuschner 2008a; Meier et al. 2018), but few highly active deep-reaching roots specialised into water uptake may have a major importance for the trees water supply.

During autumn, fine root growth usually decreases (Radville et al. 2016). However, growth flushes can still occur if environmental conditions allow it, as originally proven for various broadleaved tree species including European beech by Resa (1877). A second peak during autumn can happen even though these are usually lower than early summer growth peaks (Mainiero et al. 2010). Withington et al. (2020) found high variability of temperate tree species in the timing of root production peaks across years, with latest peaks in October, suggesting a strong influence of environmental conditions. In our study, autumn was relatively warm, mean temperatures of October 2021 were 3 °C above long-term means (Haylock et al. 2008). High temperatures in combination with increased soil moisture during autumn may have promoted root growth, thereby explaining increased root-length density in our dataset during the start of the winter. For seedlings of European broadleaved tree species, root growth decreases rapidly at temperatures below 7 °C (Schenker et al. 2014), but growth of European beech fine roots stops only when temperatures fall below 2.5 °C (Schenker et al. 2014). Even though root-length density did not change significantly between start and end of the winter, it is possible that root growth continued during winter. If so, however, winter growth rates remained in balance with decay rates.

### Ecological implications

During winter, nutrient and water uptake is negligible. Trees will therefore benefit from high fine root length mainly during the growing season. On the one hand, high root-length density during late winter can be interpreted as a smart strategy aiming to maximise root length when the season of highest demand starts. However, if these roots are already produced during the previous autumn, there is a certain risk of significant damage if the newly formed fine roots in the upper soil layers are exposed to severe soil frost. Single soil frost events are expected to increase in frequency because of reduced presence of insulting snow cover as a result of climate change (Groffman et al. 2001). Still, we detected hardly any potentially harmful soil frost events at the eight sites over the two observed winters and previous studies at the same sites (Weigel et al. 2021).

The low fine root length during summer without a trend towards deeper rooting could be an alarming sign if it was indeed a consequence of stressful conditions caused by water deficit. Due to climate warming, summer drought in temperate ecosystems is expected to become more frequent and severe in the future (IPCC 2021). However, the trees seem to be able to compensate for early season root loss during more favourable times of the year, e. g. warm and moist periods in late autumn.

Xylogenesis dynamics commonly show the end of radial growth during early August, whilst leaf senescence does not start before October in European beech (Michelot et al. 2012; Puchałka et al. 2024). So far, we assumed that the primary production during that time goes into non-structural carbohydrates (NSC) to enable leaf sprouting and early growth during the next spring. Our study suggests that the carbohydrates that are produced when radial tree growth has already stopped are potentially also invested into root growth.

### Methodological considerations

We used a recently developed root detection model to determine root-length density of European beech in a large dataset of more than 800 root images. Since the surprising results of our data raised the question whether this was an artefact of potentially higher root detection probability in response to higher soil moisture, we conducted an additional experiment to quantify this possibility. We could show that root detection probability of the RootDetector was not influenced by soil moisture. This confirms not only the general quality of the RootDetector, but also the validity of our results.

The big benefit of the automized RootDetector is the possibility to analyse large datasets with relatively little effort. However, the RootDetector provides only absolute root-length density values and does not allow for a differentiation between newly grown roots and root decay so far. Furthermore, we cannot be sure that the root-length pattern would be observed for root biomass as well, as specific root length may also differ between seasons. For further interpretation of the data, it would be highly beneficial to back up the results with destructive methods such as soil cores or ingrowth-cores, as this would allow for differentiation between alive and dead but not yet decomposed roots (e.g. by TTC staining; Comas et al. 2000), which is not accounted for with the minirhizotron method.

Considering the little effort required for image analysis with the RootDetector, further research should increase the frequency of measurements. A higher temporal resolution would allow for deeper interpretation of the root-length pattern. Previous studies show that time intervals of two to four weeks would be ideal (Mainiero et al. 2010; Withington et al. 2020). However, this might be accompanied by a decrease in the number of monitoring sites due to practical constraints. A main insight from our study is that we observed the same temporal pattern between climatically quite different monitoring sites all across the north-eastern distribution range of European beech. This study may therefore indicate that it is possible to draw generalizable conclusions even from single or few sites.

## Conclusion

Root-length density of European beech was higher at the start (+ 40%) and the end (+ 51%) of winter than in mid-summer during 2 consecutive years at eight sites across the species’ north-eastern distribution range. This finding proves the high intra-annual variability in root length of European beech. A reduction of root-length density during adverse conditions seems to be compensated afterwards during more favourable periods of the year. The lack of a trend towards deeper root growth over the dry periods, however, might be a worrying signal. A profound understanding of the seasonality of fine root dynamics is crucial for modelling terrestrial biogeochemical processes and global carbon fluxes. Our study indicates that they are more variable and, so far, less predictable than previously assumed.

## Supplementary Information

Below is the link to the electronic supplementary material.

Supplementary file1 (PDF 536 KB)

## Data Availability

All data that support the findings of this study are available from the corresponding author upon request.
