# Supplementary material for: Seasonal dynamics of fine root length in European beech: unveiling unexpected winter peaks and summer declines
Source: Oecologia. 2025 Feb 7;207(2):31. doi: 10.1007/s00442-025-05670-y (PMC11805861; doi:10.1007/s00442-025-05670-y)
Supplement: Supplementary file 1 — Supplementary file1 (PDF 536 KB) [file 442_2025_5670_MOESM1_ESM.pdf]

Electronic Supplemental Material for:

Seasonal dynamics of fine root length in European beech: unveiling unexpected winter peaks and summer declines

Aron Garthen[a], aron.garthen@uni-greifswald.de, Kirsten Brandt[a], Marcin Klisz[b], Andrey V. Malyshev[a], Bo Peters[a], Robert Weigel[c,d], Jürgen Kreyling[a]

[a] Experimental Plant Ecology, Institute of Botany and Landscape Ecology, University of Greifswald, 17489 Greifswald, Germany; [b] Department of Silviculture and Genetics of Forest Trees, Forest Research Institute, 05-090 Raszyn, Poland; [c] Ecological-Botanical Garden, University of Bayreuth, 95447 Bayreuth, Germany; [d] Plant Ecology, University of Goettingen, 37073 Goettingen, Germany

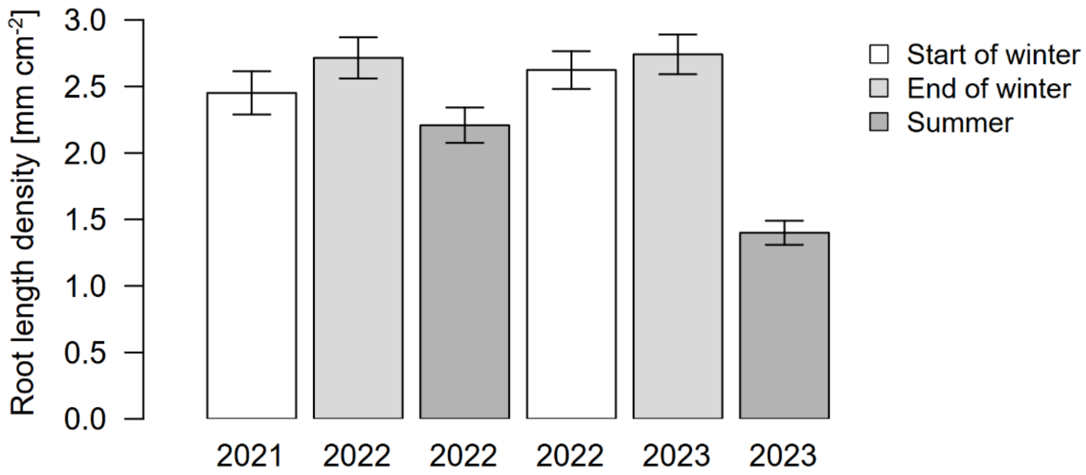

ESM1 Root length density in mm per cm² of minirhizotron scans for each of the six measurements between autumn 2021 and summer 2023 across eight forest sites between Rostock and Gdansk, quantified by the AI RootDetector. Shown are mean values and standard errors.

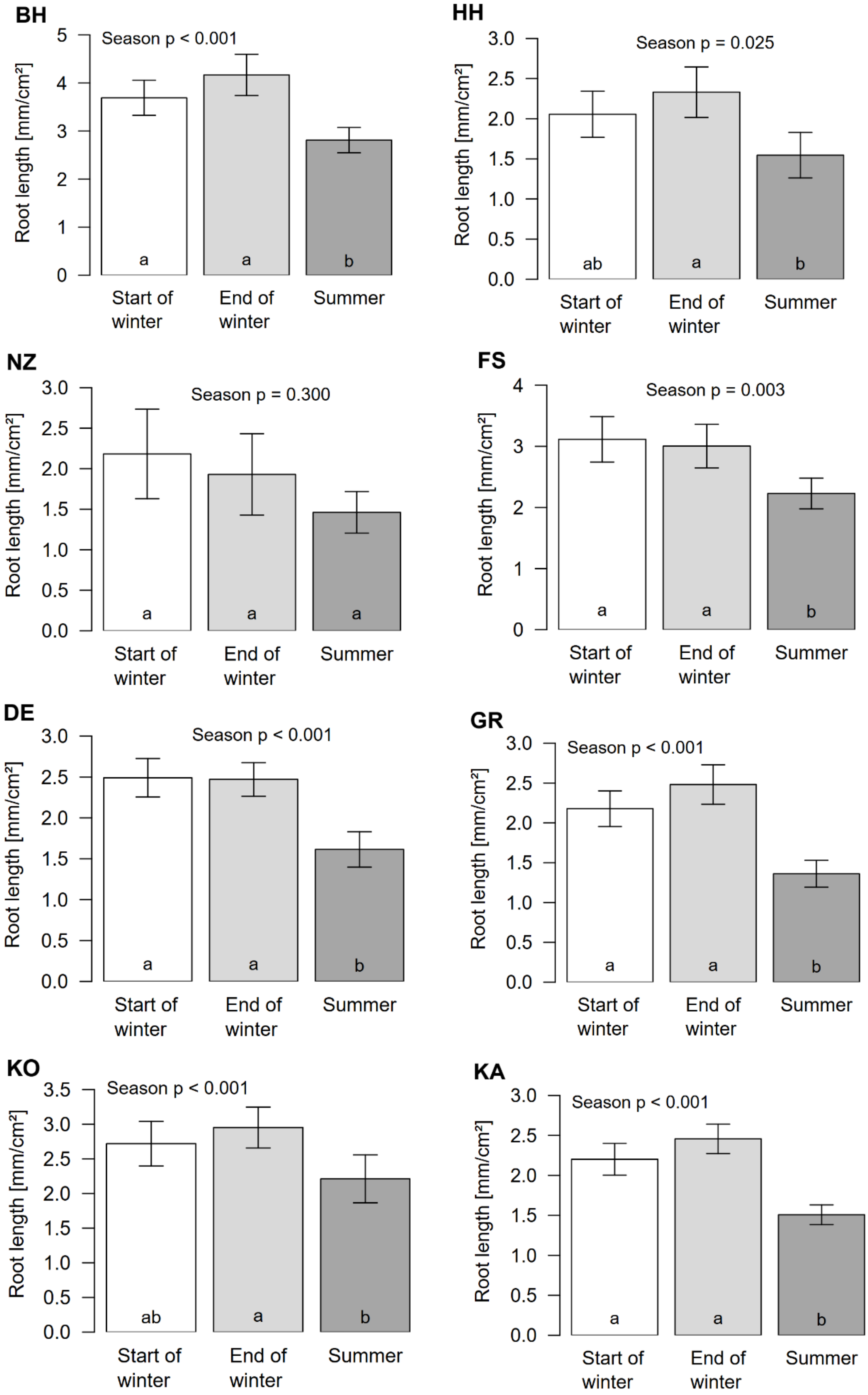

ESM2 Root length density in mm cm^{-2} of minirhizotron scans in different seasons between autumn 2021 and summer 2023 for each of the eight forest sites. Shown are mean values and standard errors. The letters displayed in the bars result from the mixed model ANOVA with subsequent pairwise comparison of estimated marginal means.

ESM3 Precipitation variables [mm] characterizing the study sites (Haylock *et al.*, 2008). Longterm data refer to the period 1991-2020.

Site	Longterm annual preci-pitation	Precipitation sum study year 2021/22	Precipitation sum study year 2022/23	Longterm precipipitation Apr – Jun	Precipitation Apr – Jun 2022	Precipitation Apr – Jun 2023
BH	628.8	518.2	590.2	153.3	92.8	58.4
HH	582.6	494.4	575.9	141.9	119.3	72.1
NZ	585.7	505.5	598.8	145.7	80.2	109.7
FS	526.4	430.4	512.9	131.7	87.3	105.3
GR	548.0	463.3	505.1	140.6	86.3	87.4
DE	518.2	401.0	576.4	130.6	77.8	129.0
KA	650.1	532.2	572.2	157.5	108.2	82.5
KO	619.2	497.3	569.6	147.7	93.8	79.90

ESM4 Temperature variables [°C] characterizing the winter month (Dec – Feb) at the study sites (Haylock *et al.*, 2008). Longterm data refer to the period 1991-2020.

Site	Avg. longterm winter temp.	Winter temp. 2021/22	Winter temp. 2022/23	Longterm coldest month temp.	Coldest month temp. 2021/22	Coldest month temp. 2022/23
BH	1.65	3.40	2.78	-0.27	1.77	0.99
DE	1.06	2.98	2.63	-1.22	1.22	1.36
FS	1.03	2.81	2.40	-1.17	0.97	1.08
GR	1.01	2.71	2.45	-1.24	0.81	1.20
HH	1.40	3.10	2.53	-0.59	1.49	0.93
KA	-1.01	0.79	0.82	-3.41	-1.26	-0.64
KO	-0.53	0.92	0.98	-2.87	-1.15	-0.43
NZ	1.11	2.82	2.44	-0.98	1.09	0.97

ESM5 List of R-packages used for statistical analyses.

Package	Reference
emmeans	Lenth R (2023) emmeans: Estimated Marginal Means. aka Least-Squares Means. R package version 1.8.5. Retrieved from https://CRAN.R-project.org/package=emmeans.
ggplot2	Wickham H (2016) ggplot2: Elegant Graphics for Data Analysis. Springer New York.
ggspatial	Dunnington D (2023) ggspatial: Spatial Data Framework for ggplot2. R package version 1.1 .9. Retrieved from https://CRAN.R-project.org/package=ggspatial.
lmerTest	Kuznetsova A, Brockhoff PB, Christensen RHB (2017) lmerTest Package: Tests in Linear Mixed Effects Models. Journal of Statistical Software 82.
multcomp	Hothorn T, Bretz F, Westfall P (2008) Simultaneous inference in general parametric models. Biom J 50:346–363.
rnaturalearth	Massicotte P, South A (2023) rnaturalearth: World map data from natural earth. R package version 1.0.1. Retrieved from https://CRAN.R-project.org/package=rnaturalearth.
rnaturalearthhires	South A, Michael S, Massicotte P (2024) rnaturalearthhires: High resolution world vector map data from natural earth used in rnaturalearth. R package version 1.0.0.9000. Retrieved from https://github.com/ropensci/rnaturalearthhires.
sciplot	Morales M, Team wcdbtRDC, community wgaftRl, Murdoch eD (2020) sciplot: Scientific graphing functions for factorial designs. R package version 1.2-0. Retrieved from https://CRAN.R-project.org/package=sciplot.
sf	Pebesma E (2018) Simple Features for R: Standardized Support for Spatial Vector Data. The R Journal 10:439-446.
